# Diversity of endosymbionts in camellia spiny whitefly, *Aleurocanthus camelliae* (Hemiptera: Aleyrodidae), estimated by 16S rRNA analysis and their biological implications

**DOI:** 10.3389/fmicb.2023.1124386

**Published:** 2023-04-17

**Authors:** Yanni Tan, Bing Gong, Qiuqiu Zhang, Changkun Li, Junyi Weng, Xia Zhou, Linhong Jin

**Affiliations:** National Key Laboratory of Green Pesticide, Key Laboratory of Green Pesticide and Agricultural Bioengineering, Ministry of Education, Center for Research and Development of Fine Chemicals, Guizhou University, Guiyang, China

**Keywords:** *Aleurocanthus camelliae*, 16S rRNA, symbiotic bacteria, rifampicin, age-stage two-sex life table

## Abstract

Camellia spiny whitefly, *Aleurocanthus camelliae* (Hemiptera: Aleyrodidae), is a major pest in tea, which poses a serious threat to tea production. Similar to many insects, various bacterial symbioses inside *A. camelliae* may participate in the reproduction, metabolism, and detoxification of the host. However, few reports included research on the microbial composition and influence on *A. camelliae* growth. We first applied high-throughput sequencing of the V4 region in the 16S rRNA of symbiotic bacteria to study its component and effect on the biological trait of *A. camelliae* by comparing it with the antibiotic treatment group. The population parameters, survival rate, and fecundity rate of *A. camelliae* were also analyzed using the age–stage two-sex life table. Our results demonstrated that phylum Proteobacteria (higher than 96.15%) dominated the whole life cycle of *A. camelliae*. It unveiled the presence of *Candidatus Portiera* (primary endosymbiont) (67.15–73.33%), *Arsenophonus* (5.58–22.89%)*, Wolbachia* (4.53–11.58%), *Rickettsia* (0.75–2.59%), and *Pseudomonas* (0.99–1.88%) genus. Antibiotic treatment caused a significant decrease in the endosymbiont, which negatively affected the host's biological properties and life process. For example, 1.5% rifampicin treatment caused a longer preadult stage in the offspring generation (55.92 d) compared to the control (49.75d) and a lower survival rate (0.36) than the control (0.60). The decreased intrinsic rate of increase (*r*), net reproductive rate (*R*_0_), and prolonged mean generation time (*T*) were signs of all disadvantageous effects associated with symbiotic reduction. Our findings confirmed the composition and richness of symbiotic bacteria in larva and adult of *A. camelliae* by an Illumina NovaSeq 6000 analysis and their influence on the development of the host by demographic research. Together, the results suggested that symbiotic bacteria play an important role in manipulating the biological development of their hosts, which might help us for developing new pest control agents and technologies for better management of *A. camelliae*.

## 1. Introduction

**Figure 1:**
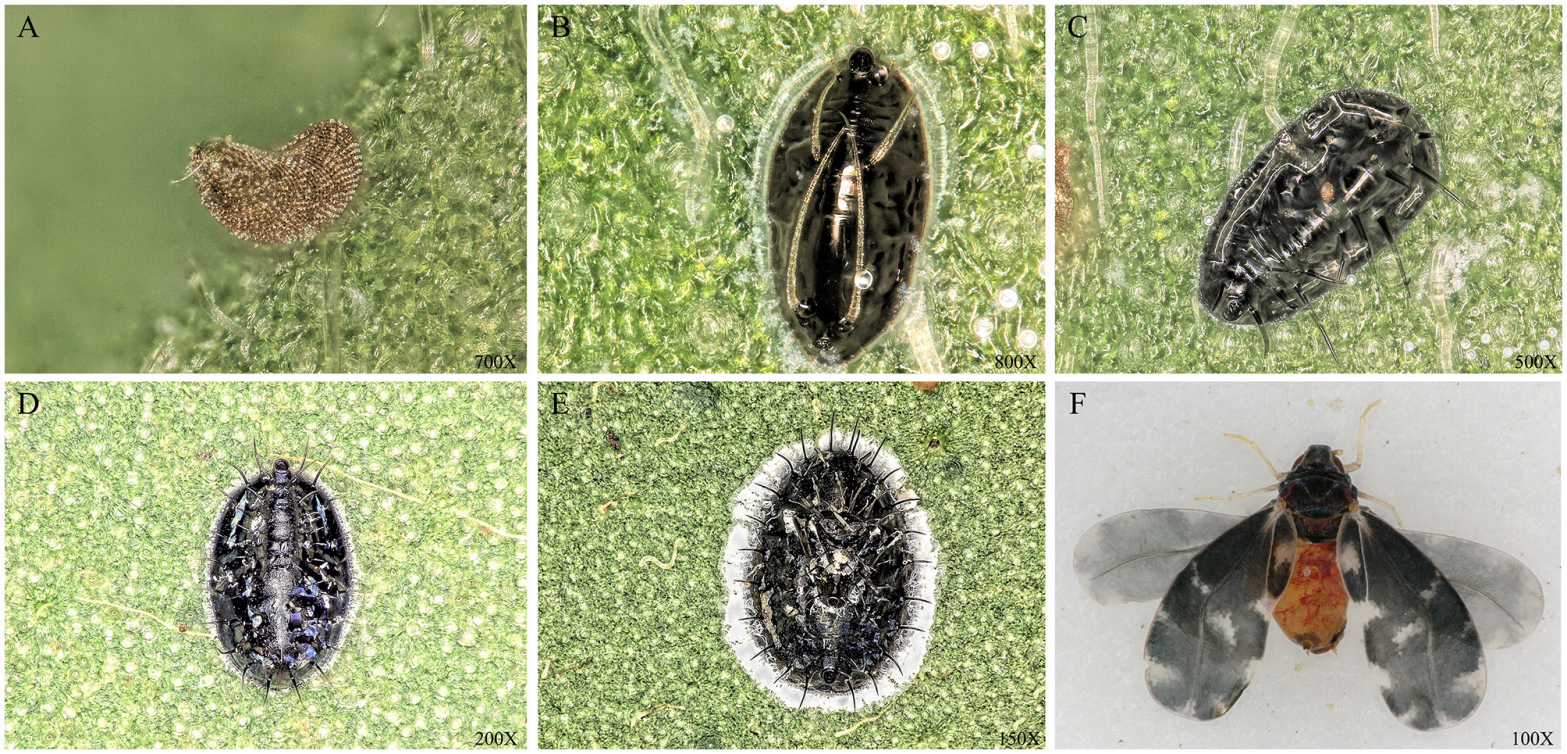
Morphology of *A. camelliae* at different stages. **(A)** egg; **(B)** the first instar nymph; **(C)** the second instar nymph; **(D)** the third instar nymph; **(E)** Pupa; and **(F)** Adult. The picture was taken under the Ultra-Depth Microscope (Leica DVM5, Germany).

The Camellia spiny whitefly, *Aleurocanthus camelliae* (Hemiptera: Aleyrodidae) (Figure 1), is a major pest to the Theaceae plants that originated in China (Kanmiya et al., 2011; Chen et al., 2018; Andrianto and Kasai, 2022) and is currently considered to be an invasive species over the world tea producing area (Uesugi et al., 2016; Jansen and Porcelli, 2018; Adi and Susanti, 2020; Rizzo et al., 2021). The main harm caused by the *A. camelliae* whitefly is the sucking of tea sap and the excretion of honeydew that covers the surfaces of tea leaves which subsequently promotes the growth of sooty mold. The combination of all these factors causes a decline in the health and vigor of the tea tree and a reduction in yield (Tian et al., 2020). In fact, citrus whitefly *A. spiniferus* had been denoted as *A. camelliae* before 2011 till the Japanese researchers proved the difference between the spiny whitefly on tea trees and the whitefly *A. spiniferus* on citrus and defined the new scientific name of *Aleurocanthus camelliae* for Camellia spiny whitefly in tea plant (Kanmiya et al., 2011; Uesugi and Sato, 2011).

Insects generally carry heritable symbiotic bacteria including primary and secondary symbionts which coevolve with host insects (Wang et al., 2020), and endosymbionts play an important role in insect growth and reproduction (Hillman et al., 2017; Akami et al., 2019; Zhang et al., 2019). Cooperation between the host insect and its microbiota is largely beneficial for all partners, but shifts in microbiota composition can also violate this mutualism (dysbiosis) and result in severe adverse effects on insects (Engl and Kaltenpoth, 2018; Grenier and Leulier, 2020). It is known that *Aleyrodidae* species harbor endosymbiont *Bacteroides* playing fundamental roles in their fitness. In addition, it has been demonstrated that endosymbionts can both protect their host against enemies (i.e., parasitoids, other bacteria, and viruses) and influence some biological traits (e.g., fitness, sex ratio, and host range), usually ameliorating the growth rate of the host under certain stress conditions (Bing et al., 2013; Su et al., 2014; Bubici et al., 2020). Limited studies related to bacterial symbionts of other close species have been examined, including *A. woglumi* (Pandey et al., 2013) and *A. spiniferus* (Bubici et al., 2020). However, bacterial symbionts of *A. camelliae* are poorly understood except for a report on the *Wolbachia* infection in *Aleurocanthus* cryptic species complex (Andrianto and Kasai, 2022). Therefore, this study aimed to examine the infection status and diversity of symbionts and their influence on *A. camelliae*.

Many 16S rRNA-based surveys of intestinal microbiota use taxonomic classification as the primary tool for comparative analysis to identify taxa that are indicative of a certain host phenotype. For example, 16S rRNA gene sequencing reveals the diversity and dynamic change of bacterial symbionts of *Adelphocoris suturalis* (Xue et al., 2021) and a shift in the microbiota of *Diaphorina citri* during the psyllid life cycle (Meng et al., 2019) and environmental-associated microbiota composition in honey bees (Jones et al., 2018). However, there was a lack of systematic research on the microorganisms hosted in *A. camelliae* and their function. We, therefore, by using high throughput sequencing of the bacterial 16S rRNA genes, detected the composition and diversity of bacteria in the *A. camelliae* to grasp the resource status of bacterial microorganisms in this whitefly.

The functions of the symbionts in whiteflies have attracted increasing research efforts in recent years. For example, the *Cardinium* provides protection of *Bemisia tabaci* against heat stress and can increase the longevity of both female and male adults and oviposition periods (Yang et al., 2021). The abundance of the *Portiera* in the whitefly population decreased with the increase in the nitrogen levels of the host plant (Liu et al., 2020). *Hamiltonella*–*Cardinium* coinfection induced lower fecundity, egg hatchability, and a number of female offspring, leading to a male-biased sex ratio in *B. tabaci* (Zhao et al., 2018). In addition, *Hamiltonella* elimination from the whitefly host by heat treatment and antibiotics influences the sex ratio of an insect host (Shan et al., 2019). Symbiotic bacteria of whitefly together affected host resistance to neonicotinoids (Barman et al., 2022). Reductions of primary and secondary symbionts in whiteflies following an antibiotic treatment negatively affect the host's fitness (Zhang et al., 2015).

Because most of the bacterial symbionts found in whiteflies and other sap-sucking insects are uncultivable, one way to study the functions of these symbionts is to establish host lines that differ in symbiosis treatment and then compare the performance between different host lines. For this purpose, antibiotic treatment of the hosts has frequently been employed to cure symbionts (Zhang et al., 2020; Weiland et al., 2022). This approach has also been tried with whiteflies in *B. tabaci* (Shan et al., 2016; Lv et al., 2018; Andreason et al., 2020). The antibiotic rifampicin was used in the experiments because all the previous articles, which present successful elimination or suppression of whitefly symbionts (Shan et al., 2016; Zhao et al., 2020), report that rifampicin was the most selective and efficient agent in their experiments between the several antibiotics tested, including tetracycline and ampicillin. In this study, we examined the effects of rifampicin treatments on the demographic development of the Camellia whitefly and its symbionts, including the primary symbiont *Portiera* and other secondary symbionts.

Life table analysis is the most widely used method to study life history characteristics and population dynamics, which can provide a powerful tool to describe the impact of external factors on the growth, development, reproductive capacity, and population growth of insects (Chi et al., 2020; Yang et al., 2020). However, the demographic evaluation of *A. camelliae* and its changes caused by symbionts have not been studied so far. This research on symbionts of *A. camelliae* revealed the endosymbionts and their function on host reproduction, survival, population dynamics, and adaptation to the environment. It will provide new insight into the biological function of endosymbionts and the theoretical basis for the potential biological control of *A. camelliae*.

## 2. Materials and methods

### 2.1. Insect rearing and maintenance

The *A. camelliae* individuals used in this study were collected from a tea garden in Qingzhen (N 26°55′, E 106°47′), Guizhou, China. The population was maintained on 2-year tea plants at 25 ± 1°C, 70 ± 5% relative humidity, and a photoperiod of 14:10 h (L:D) in climatic chambers, and the insects are raised for more than two generations for research. Tea plants with 6–8 true-leaf stages were selected for consumption in experiments. Molecular identification of the mtDNA COI gene and morphological observation to study the species identification of *A. camelliae* were referred to in previous studies (Chen et al., 2018).

### 2.2. Diversity analysis of symbiotic bacteria in *A. camelliae*

#### 2.2.1. Sampling and DNA extraction

All *A. camelliae* at the third larva and 1-day-old adult stage were starved for 6 h and then were surface sterilized with 75% ethanol for 2 min and rinsed with distilled sterile water three times before DNA extraction. Insects were classified as larva (Ac. L) and adult (Ac. A) with three duplicates for each group. Total bacterial DNAs of *A. camelliae* were isolated from 50 individuals using the Insect DNA Isolation kit (Omega, United States), according to the manufacturer's instructions, and stored at −80°C. Before library construction, the concentration and integrality of all DNA samples were analyzed by microspectrophotometer (Implen, Germany) and agarose gel electrophoresis.

#### 2.2.2. PCR amplification and sequence data processing

The V4 region of bacterial 16S rRNA genes was amplified from the DNA extracts using the bacterial-specific primers (V4-515F: 5' -GTGCCAGCMGCCGCGGTAA−3', V4-806R: 5'-GGACTACHVGGGTWTCTAAT-3'). PCR enrichment was performed in a 30 μl reaction containing a 10 μl template, a fusion PCR primer, and a PCR master mix. PCR cycling conditions were as follows: 98°C for 1 min, 30 cycles of 98°C for 10 s, 50°C for 30 s, 72°C for 30 s, and a final extension at 72°C for 5 min. After validation by 2% agarose gel electrophoresis, PCR products were purified using a Gel Extraction kit (Qiagen, Germany) and then pooled in equimolar concentrations. Sequencing libraries were generated using TruSeq^®^ DNA PCR-Free Sample Preparation kit (Illumina, United States), following the manufacturer's recommendations and qualified by the Agilent 5400 bioanalyzer (Agilent, United States). The validated libraries were used for sequencing on an Illumina NovaSeq 6000 platform (Illumina, United States), according to the standard Illumina procedures.

### 2.3. Symbiotic reduction of *A. camelliae* by antibiotic treatment

#### 2.3.1. Symbiotic reduction

We used plant-mediated antibiotic delivery methods to obtain aposymbiotic whitefly samples by treating the natural adult *A. camelliae*, according to the previous report (Ayoubi et al., 2020), and minor improvement. Rifampicin was applied as an antibiotic agent and resolved in 2% glycerol to prepare a 1.5% antibiotic solution. Each 15 ml rifampicin (1.5%) solution was sprayed onto a tea plant with 6–8 leaves. After the solution on the surface of the leaves was completely dry (~5 h), the *A. camelliae* adults were subjected to each plant. In addition, the eggs (F1) produced by these adults in 24 h were kept on the plant for the following observation (the parental adults were cleared). In total, a 2% glycerol solution served as the control, and six repetitions were set for the control and rifampicin treatment (aposymbiotic) groups.

#### 2.3.2. DNA extraction and real-time quantitative PCR (qPCR)

DNA from control and symbionts-suppressed treatments of insects were obtained by the same procedure as described earlier. Extraction samples were subjected to real-time quantitative PCR (qPCR) to check the bacterial content and change by using the specific primers to each endosymbiont as presented in Table 1 with the β*-Actin* gene serving as the selected reference gene. The PCR reaction mixture was prepared as follows: 10 μl of 2× Real Star Green Fast Mixture (GenStar, China), 0.5 μl of Primer F and Primer R, 1μl of DNA template, and proper ddH_2_O were added to achieve a 20 μl final reaction solution. The reaction mixture was initially denatured at 95°C for 120 s, followed by 40 cycles of amplification at 95°C for 15 s, 60°C for 30 s, and 72°C for 30 s using a Light Cycler^®^96 (Roche, Switzerland). qPCR data were analyzed using the 2^−Δ*Ct*^ method (Livak and Schmittgen, 2001), and the relative quantity of symbionts was achieved.

**Table 1:** Primers used in this real-time quantitative PCR.

**Target species**	**Primer name**	**Primer sequence(5' → 3')**	**Product size(bp)**	**Reference**
*β-Actin*	Actin-F	TCTTCCAGCCATCCTTCTTG	130	Ren et al., 2021
	Actin-R	CGGTGATTTCCTTCTGCATT		
*Portiera*	Port73-F	GTGGGGAATAACGTACGG	200	Shan et al., 2016
	Port266-R	CTCAGTCCCAGTGTGGCTG		
*Arsenophonus*	A-23s-F2	ATGGTGCCGTAACTTCAGGA	174	Ren et al., 2021
	A-23s-R2	TAACCTTACAGCACCTGGCA		
*Wolbachia*	wspQ384-F	TGGAACCCGCTGTGAATGAT	130	Zhou, 2018
	wspQ513-R	GCACCATAAGAACCGAAATAACG		
*Rickettsia*	glt375-F	TGGTATTGCATCGCTTTGGG	200	Caspi-Fluger et al., 2011
	glt574-R	TTTCTTTAAGCACTGCAGCACG		
*Pseudomonas*	gyrB8-1-F	TGCGGTCAACCAGGTGTTCC	271	Min et al., 2020
	gyrB8-1-R	CGAGATAATCGCGGTCAGG		

### 2.4. Life table study of *A. camelliae* compared with aposymbiotic treatment

*Aleurocanthus camelliae* pairs that had enclosed within 12 h of the collection were placed in a cage (50 cm × 50 cm × 50 cm) with 2-year tea seedlings for both control and aposymbiotic treatments as described earlier. After 24 h egg-laying on the leaves, 100 eggs were kept in each group, and the excess eggs and adult pairs were cleared. The tea seedlings with the eggs of *A. camelliae* were kept in climatic chambers at 25 ± 1°C, 70 ± 5% RH, and 14:10 h (L:D).

Once hatched, the first instar nymphs will settle on the leaf underside and stay till their pupa stage (the immature stages of *A. camelliae* begin life as mobile individuals but soon attach to host plants), so the positions of the whole nymph stage could be photographically marked and recorded. Their developmental duration and survival were then daily observed during the whole generation. After the emergence of adult *A. camelliae*, each pair of them was switched to the rear on a fresh tea shoot in an isolated insect box (20 × 25 × 10 cm). Fresh new tea shoots were replaced daily for adults to count the amount of newly laid eggs until all the adults died.

### 2.5. Data static analysis

#### 2.5.1. Sequencing analysis of bacterial symbionts

The data obtained from independent sequencing were analyzed separately. Replicate samples of the third instar larva (Ac. L 1/2/3) and 1-day-old adult (Ac. A 1/2/3) were included in this study. Raw reads were filtered to remove adaptors, and low-quality and ambiguous bases and paired-end reads were added to tags using the Fast Length Adjustment of Short reads program (Magoc and Salzberg, 2011) to obtain the tags. The Uparse (Edgar, 2013) algorithm was used to cluster all effective tags of all samples, and a nucleotide similarity threshold of 97% was used to assign sequence reads to operational taxonomic units (OTUs). Taxonomic information was annotated for representative sequences of each OTU by using the Mothur method (Schloss, 2020) and SSU rRNA database in SILVA138.1 (Quast et al., 2013) to obtain the community composition of each sample. The Shannon, Simpson, Chao1 (Kuczynski et al., 2012), and Ace diversity indices were used to assess species diversity among samples. Alpha diversity was estimated by QIIME (Caporaso et al., 2010), and the dilution curves were drawn using R software (v 2.15.3). We calculated the abundance of each functional category based on the information in the KEGG database and the OTU abundance information. In addition, PICRUSt was used to obtain the levels of metabolic pathway information, and the abundance of each level was obtained.

#### 2.5.2. Two-sex life table analysis

The analysis of life table raw data was performed based on the age–stage, two-sex life table theory (Chi and Liu, 1984; Chi, 1988) by using the computer program, TWOSEX-MSChart. The parameters including the age–stage-specific survival rate (*s*_*xj*_), age-specific survival rate (*l*_*x*_), age–stage-specific fecundity (*f*_*xj*_), age-specific fecundity (*m*_*x*_), net maternity (*l*_*x*_*m*_*x*_), age–stage life expectancy (*e*_*xj*_), intrinsic rate of increase (*r*), finite rate of increase (λ), net reproductive rate (*R*_0_), and mean generation time (*T*) were calculated accordingly referring to the report (Khanamani et al., 2017). The age–stage-specific survival rate (*s*_*xj*_) is the probability that a newborn individual survives to age *x* and stage *j*. The age–stage-specific fecundity (*f*_*xj*_) represents the daily number of eggs produced per female of age x. The age–stage life expectancy (*e*_*xj*_) indicates the total expected survival time of individuals in age x. We also determined the adult and nymphal stage longevities, the durations of preoviposition periods including the adult preoviposition period (APOP) and total preoviposition period (TPOP), as well as the number of egg masses produced during the lifetime of the females at each treatment (fecundity). The mean and standard errors of the population parameters were calculated by using the bootstrap procedure with 100,000 resamplings.

## 3. Results

### 3.1. Diversity analysis of symbiotic bacteria in *A. camelliae*

#### 3.1.1. Pyrosequencing of bacterial 16S rRNA gene

The analysis of symbiotic bacteria composition and abundance in *A. camelliae* was analyzed by Illumina NovaSeq 6000 *via* the sequencing of the 16S rRNA gene. We obtained a total of 520,336 raw tags and 491,297 effective tags, with a mean length of 425 bp in six samples (Table 2). Based on 97% species similarity, we clustered the spliced tags into OTU. The number of OTUs at the larva and adult stages is presented in Table 2. We constructed dilution curves for Ace, Chao1, Shannon, Simpson, and Good's coverage and observed species, which demonstrated the quality and credibility of sequencing quantity (Supplementary Figure 1). Good's coverage of all samples was above 99%, indicating that our sequencing results were sufficient to fully estimate the diversity of the *A. camelliae* bacterial community (Table 2).

**Table 2:** Summary of 16S rRNA gene sequencing data.

**Sample name**	**Raw tags**	**Effective tags**	**Mean length (bp)**	**Number of OTUs**	**Chao1**	**Ace**	**Shannon**	**Simpson**	**Good's coverage**
Ac.L1	88,403	83,190	427	296	275.060	281.277	1.698	0.550	0.999
Ac.L2	93,310	88,898	427	210	218.129	220.930	1.387	0.432	0.999
Ac.L3	86,727	83,264	424	295	282.500	283.052	1.667	0.486	0.999
Ac.A1	88,828	82,922	421	311	320.442	316.493	2.005	0.562	0.999
Ac.A2	79,192	74,418	425	242	256.231	284.499	1.327	0.342	0.999
Ac.A3	83,876	78,605	424	304	288.548	303.919	1.551	0.413	0.999

Samples were named according to their developmental stage. Ac. L: 3^rd^ instar larva; Ac. A: adult eclosion at 1 day.

#### 3.1.2. Bacterial diversity in the *A. camelliae*

**Figure 2:**
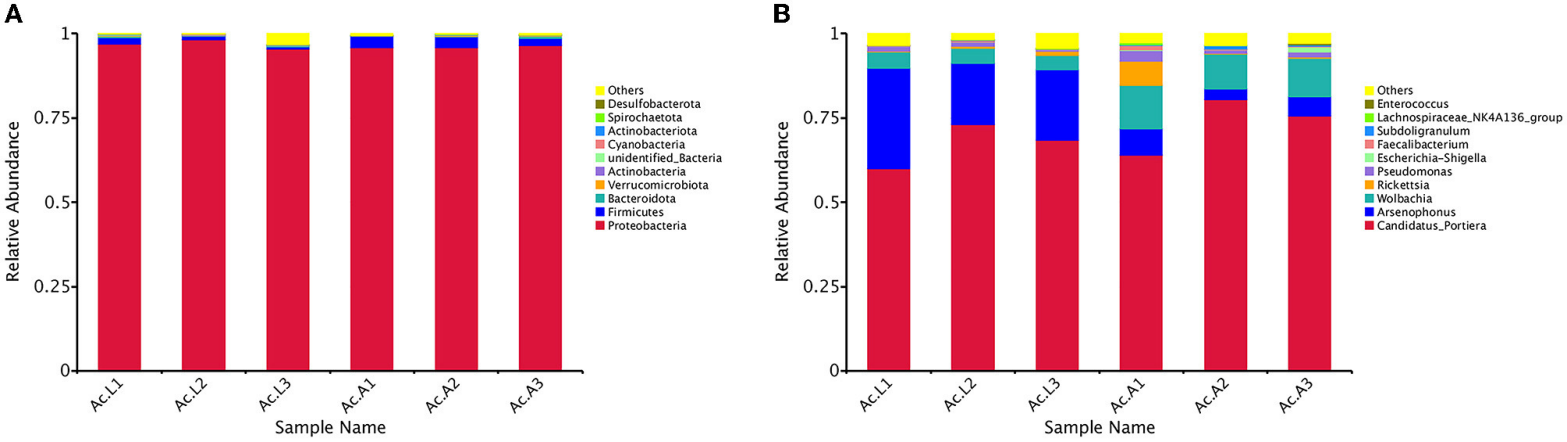
Bacterial community dynamics among larvae and adults in *A. camelliae*. **(A)** Relative abundance of bacteria communities at the phylum level in different groups. **(B)** Relative abundance of bacteria communities at the genus level in different groups.

Among all the larvae, 13 bacterial phyla, 22 classes, 50 orders, 85 families, and 117 genera were detected. However, there were 14 bacterial phyla, 29 classes, 60 orders, 95 families, and 139 genera in the adults (Table 3). The relative abundance of the 10 most abundant bacterial groups at the phylum and genus levels within each library is shown in Figure 2. The 10 most represented phyla were Proteobacteria, Firmicutes, Bacteroidota, Verrucomicrobiota, Actinobacteria, unidentified_Bacteria, Cyanobacteria, Actinobacteriota, Spirochaetota, and Desulfobacterota. Among them, the Proteobacteria were most abundant in all the libraries and comprised 96.82% and 96.15% of the bacterial communities in the larva and adult communities, respectively. Firmicutes were the next most abundant phylum and comprised 1.22% and 2.85% of the corresponding communities (Figure 2A). At the class level, the most abundant bacterial community was Gammaproteobacteria and comprising 91.40% and 81.90% of the larva and adult communities, respectively. Alphaproteobacteria and Clostridia were the next most abundant classes. At the order level, the three most abundant bacterial communities are Pseudomonadales, Enterobacterales, and Rickettsiales and represented 68.18%, 23.12%, and 5.29% of all of the OTUs in the larvae, respectively, and 75.35%, 6.51%, and 14.17% in adults, respectively. Overall, the 10 most abundant families identified were the Halomonadaceae, Morganellaceae, Anaplasmataceae, Rickettsiaceae, Pseudomonadaceae, Enterobacteriaceae, Ruminococcaceae, Lachnospiraceae, Enterococcaceae, and Hafniaceae. Halomonadaceae dominated the whole community and represented 67.15% of all OTUs in the larva community and 73.33% of those in the adult community. The Morganellaceae (22.90%) accounted second in the larva while the Anaplasmataceae (11.58%) accounted second in the adult stage (data for class, order, and family are available in Supplementary Figure 2).

**Table 3:** Bacterial community composition within *A. camelliae*.

**Sample name**	**Phylum**	**Class**	**Order**	**Family**	**Genus**
Ac. L	13	22	50	85	117
Ac. A	14	29	60	95	139

At the general level, the first 10 compositions were *Candidatus_Portiera, Arsenophonus, Wolbachia, Rickettsia, Pseudomonas, Escherichia-Shigella, Faecalibacterium, Subdoligranulum, Lachnospiraceae_NK4A136_group*, and *Enterococcus* (Figure 2B). *Candidatus_Portiera* ranked first and comprised 67.15% and 73.33% of the bacterial communities in the larva and adult communities, respectively. *Arsenophonus* was the most abundant secondary symbiont in the larva stage *A. camelliae* and accounted for 22.89%, while *Wolbachia* was the most abundant secondary symbiont in adults *A. camelliae* and accounted for 11.58%. In addition, with the growth of *A. camelliae* from larvae to adults, the quantity of secondary symbiotic bacteria *Arsenophonus* decreased and *Wolbachia* increased (Figure 2B).

**Figure 3:**
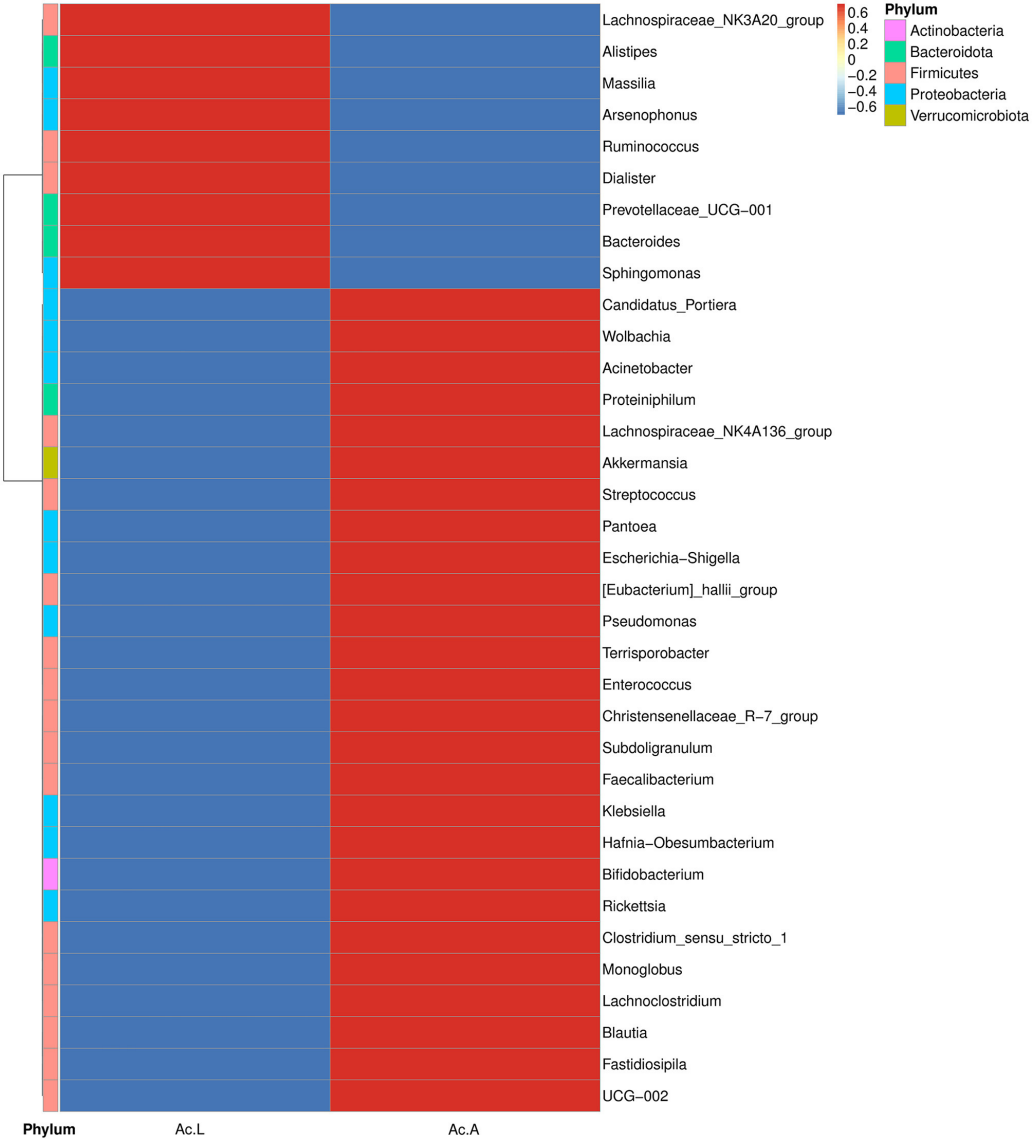
Heat map analysis of the top 35 microbial populations with relative abundance at *A. camelliae*. Ac. L and Ac. A refers to larvae and adults of *A. camelliae*, respectively.

The abundance and diversity of symbiotic bacteria are reflected through multiple indicators (Chao1, Ace, Shannon, and Simpson indices). The results showed that the abundance of symbiotic bacteria in adults was higher than that in larvae, and the diversity is not different from that of larvae (Table 2). We selected the bacterial genera with the top 35 abundance ratios and drew heat maps based on their relative abundance in larvae and adults (Figure 3). Different colors in the same row indicate the difference in abundance between larvae and adults in *A. camelliae*. The left side showed the similarity between bacteria. The analysis shows that the relative abundance of the most symbiotic bacteria in the adult stage is higher than that in the larva stage which is consistent with the species analysis results. The relative abundance of *Arsenophonus* in the larva population is higher than that in the adult population. The relative abundance of *Portiera, Wolbachia, Rickettsia*, and *Pseudomonas* in the adult population is higher than that in the larva population (Figure 3). It further indicates that the characteristics of symbiotic bacteria under different insect states are different at the two principal component levels.

### 3.2. Suppressed symbiont titer under antibiotics treatment

**Figure 4:**
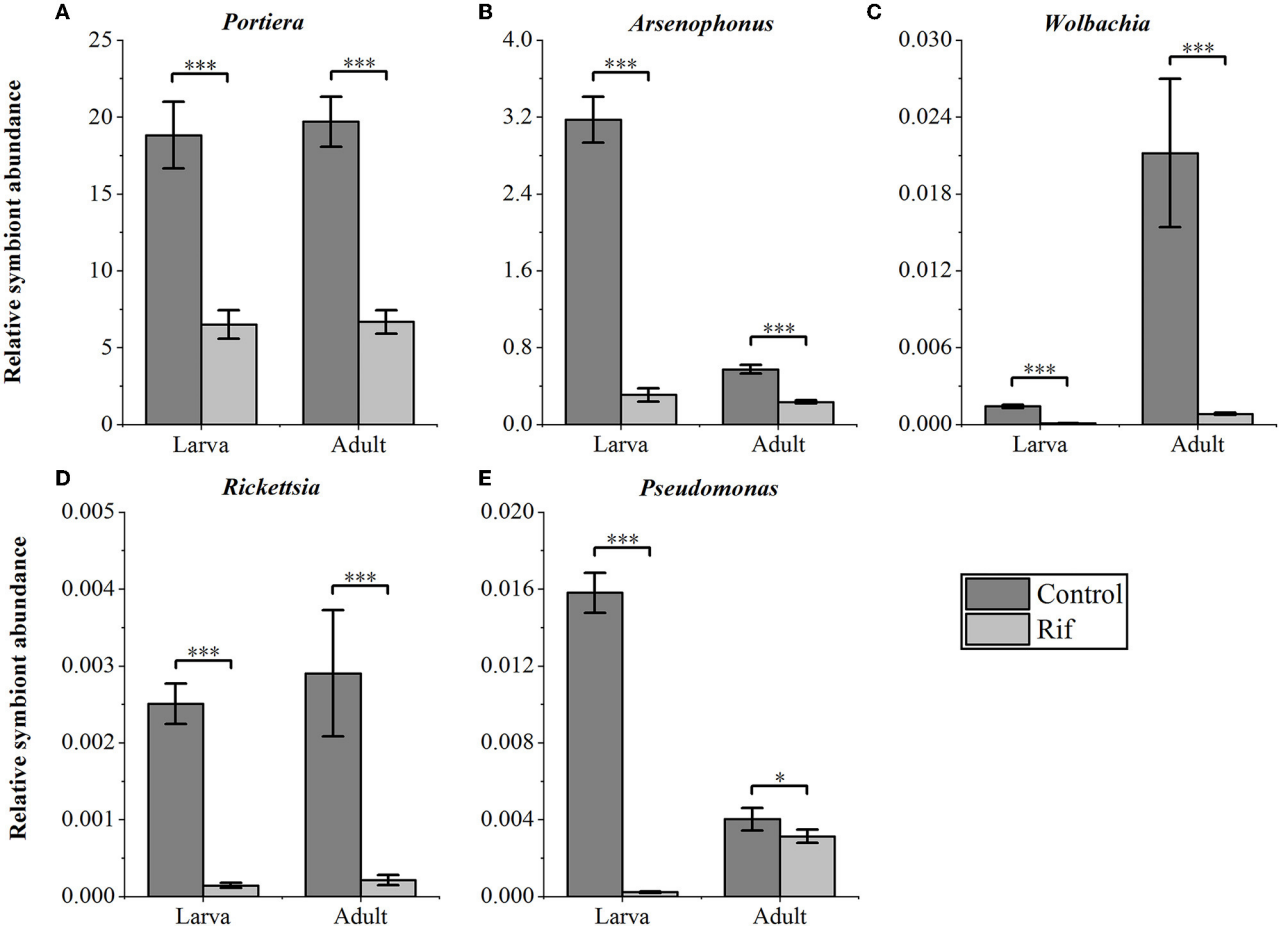
qPCR quantification of bacterial symbionts in *A. camelliae* accompanied by rifampicin (Rif) treatments in the larva (3^rd^ nymph) and adult stages. **(A)** *Portiera*, **(B)** *Arsenophonus*, **(C)** *Wolbachia*, **(D)** *Rickettsia*, and **(E)** *Pseudomonas*. The error bars indicate the standard errors (*P < 0.05, ***P < 0.001, *t*-test. *N* = 6).

We confirmed the abundance and reduction of the bacterial symbionts in *A. camelliae* by qPCR in control and plant-mediated antibiotic treatment. This bar chart compared the relative symbionts' abundance in the third nymph and adult stages in *A. camelliae* considering the aposymbiotic treatment (Figure 4). Consistent with the NovaSeq 6000 sequencing, all five targeted symbionts detected by qPCR represented the same relative density and change trend except *Wolbachia* and *Arsenophonus*. *Portiera* as the primary symbiont accounted overwhelmingly highest in both stages. For the secondary symbionts, the relative density of *Wolbachia* is lower than *Arsenophonus* in both stages by qPCR, but *Wolbachia* is higher than *Arsenophonus* in adults by 16s rRNA sequencing. The bacterial symbionts including *Portiera, Arsenophonus, Rickettsia, Wolbachia*, and *Pseudomonas* in the rif (rifampicin treatment) were all dramatically declined but not completely cleared (Figure 4). For the third nymph *A. camelliae*, the levels of *Portiera, Arsenophonus, Wolbachia, Rickettsia*, and *Pseudomonas* were ~3-, 10-, 14-, 17-, and 60-folds lower than that of the control group, respectively. For the adult stage, the symbionts were approximately 3-, 2-, 26-, 13-, and 1-folds reduced than that of the control group.

### 3.3. Life table study of *A. camelliae* and influence of rifampicin

#### 3.3.1. Development of the *A. camelliae* in different age stages

Eggs collected from rif (rifampicin treatment) and the control group were involved to check their fitness analysis including population growth parameters, duration of different stages, adult preoviposition period (APOP), total preoviposition period (TPOP), and fecundity to achieve the life table of *A. camelliae*.

The duration of the stage for egg, first to third instar nymph, and total preadult of F1 *A. camelliae* in aposymbiotic insect (rifampicin treatment) were significantly longer than those of the symbiotic insect (control), though with no significant difference in the pupal stage (Table 4). However, the TPOP of the aposymbiotic insect was ~7 days extended compared with 50.29 days in the control group (Table 4), while the following APOP had no significant difference for the two groups. The fecundity of aposymbiotic *A. camelliae* was 21.88%, and ~32.2% decreased from 31.32% offspring in the control group. In addition, the number of female and male offspring was close in the control group with 34 female and 30 male (F: M = 1.13:1) insects and changed to 24 female and 14 male (F: M = 1.71:1) insects when the symbiont was suppressed. The offspring of both sexes dropped significantly, and an obvious disequilibrium toward the dominance of females (1.71:1) happened after the symbiont was suppressed. These observations suggested that the males are more susceptible to antibiotic treatment than the females.

**Table 4:** Stages and developmental periods (days) (mean ± SE) of *A. camelliae* from the rifampicin treatment and control group.

**Life history parameters**	**Control**	**Rif**
Egg duration (d)	14.00 ± 0.06b	15.41 ± 0.11a
1st instar nymph (d)	9.25 ± 0.09b	11.74 ± 0.10a
2nd instar nymph (d)	7.05 ± 0.08b	8.33 ± 0.09a
3rd instar nymph (d)	7.81 ± 0.07b	8.91 ± 0.08a
Pupal stage (d)	11.86 ± 0.13a	12.18 ± 0.11a
Total preadult duration (d)	49.75 ± 0.22b	55.92 ± 0.24a
Total preoviposition period (TPOP) (d)	50.29 ± 0.30b	57.00 ± 0.26a
Adult preoviposition period (APOP) (d)	0.27 ± 0.07a	0.38 ± 0.10a
Adult longevity (d)	5.78 ± 0.10a	4.74 ± 0.13b
Fecundity (F) (No. of eggs per female)	31.32 ± 1.37a	21.88 ± 1.24b
Sex ration (F:M)	1.13 (34:30)	1.71 (24:14)

Standard errors were estimated by using 100,000 resampling. Means followed by different letters are significantly different according to paired bootstrap test based on the confidence intervals of differences between treatments (*P* < 0.05).

#### 3.3.2. Survival and reproduction rate of the *A. camelliae*

**Figure 5:**
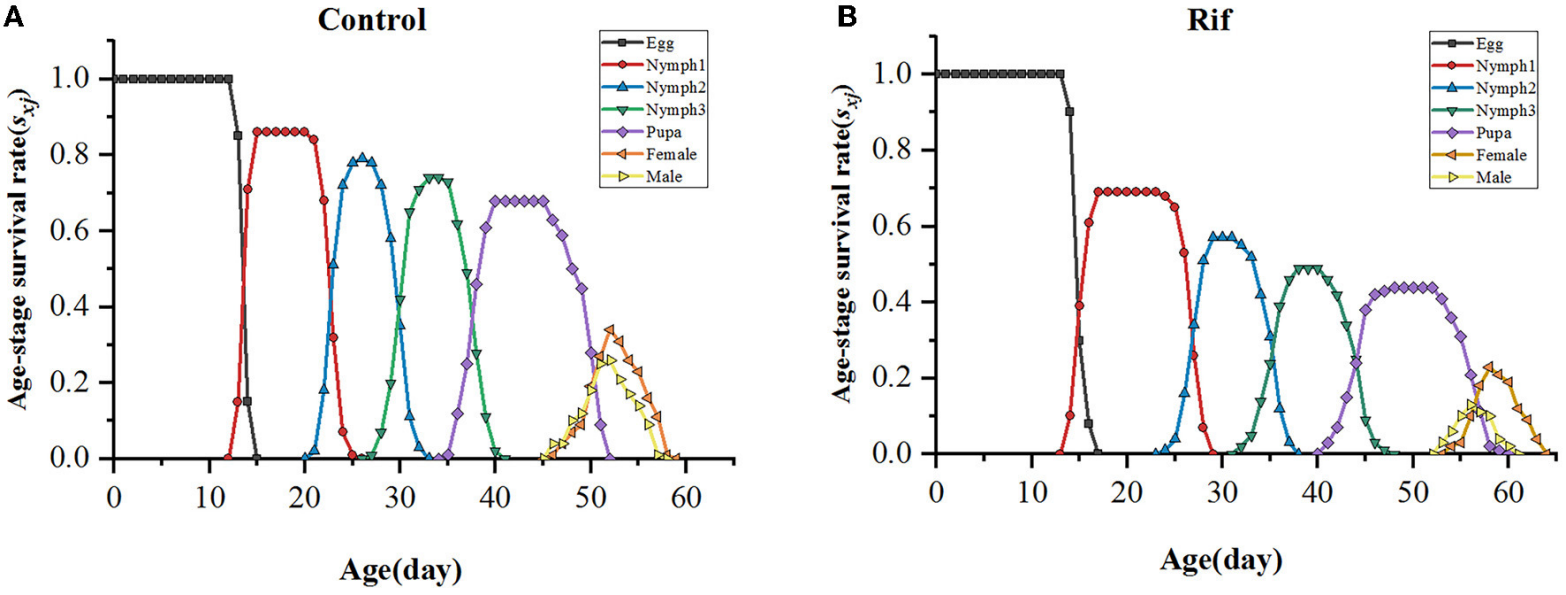
Age–stage-specific survival rate (*s*_*xj*_) of *A. camelliae*. **(A)** Control. **(B)** Rif-treatment.

The age–stage-specific survival rate (*s*_*xj*_) of *A. camelliae* (Figure 5) between aposymbiotic insect and control showed different survivorship and various developmental rates. The survival rate of the offspring of *A. camelliae* exposed to rifampicin decreased rapidly and was systemically lower than that of the control. The probability of the aposymbiotic insects developing from egg to first instar nymph was 0.69, which is lower than 0.86 in symbiotic insects. The second instar nymph and third instar nymph were 0.57 and 0.49, respectively, which is also lower than the survival probability of the symbiotic insect (0.78 and 0.74). Similarly, the probability for adult survival (0.36) remarkably declined to that of the symbiotic insect (0.60). This indicated that the emergence or survival rate of both females and males in the aposymbiotic whitefly was consistently lower than the natural insects. Especially, male survival was strongly eliminated resulting in sex ratios biased to females.

**Figure 6:**
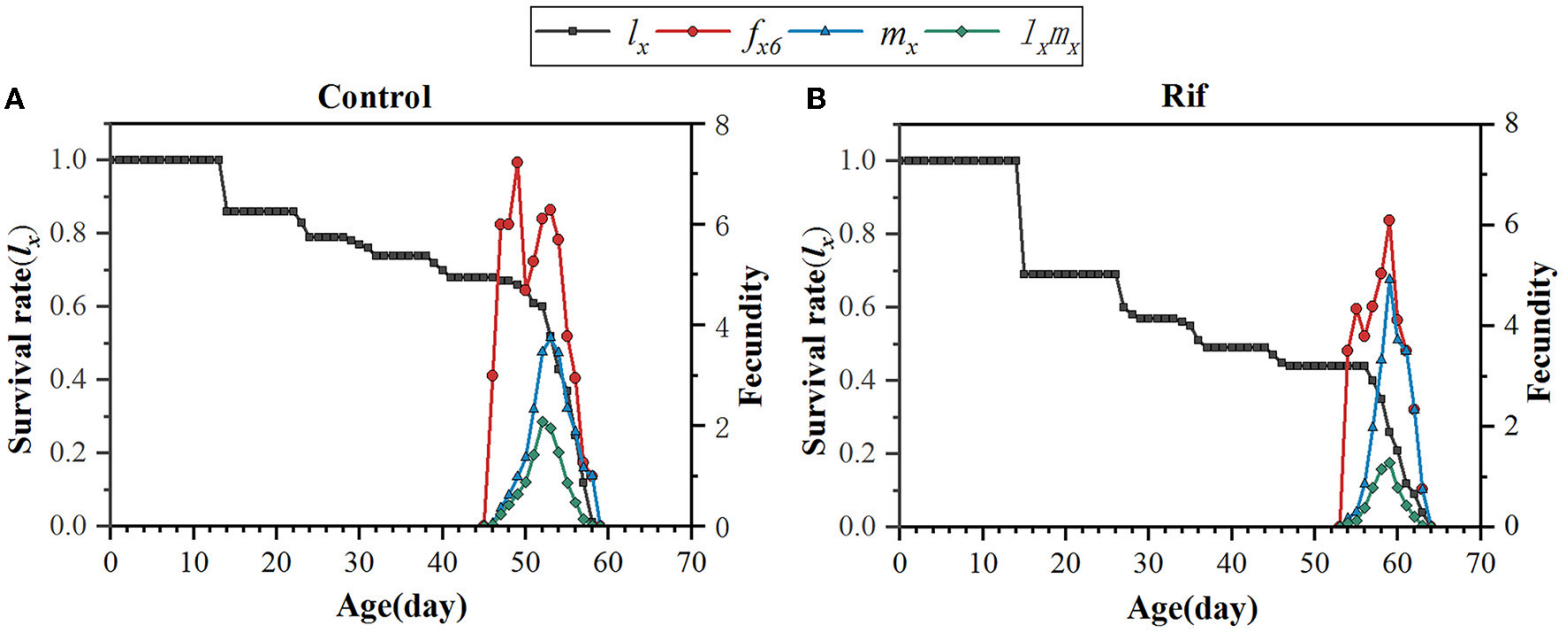
Age-specific survival rate (*l*_*x*_), age–stage-specific fecundity (*f*_*xj*_), age-specific fecundity (*m*_*x*_), and net maternity (*l*_*x*_*m*_*x*_) of *A. camelliae* in the control **(A)** and the rif-treatment **(B)**.

The probability for age-specific survival rate and fecundity was also evaluated, as shown in Figure 6. The age-specific survival rate (*l*_*x*_) shows the potentiality that a newborn individual will survive to age x and is calculated by pooling all individuals of both sexes. Therefore, the curve of *l*_*x*_ is a simplified version of the *s*_*xj*_ curve, which is presented in Figure 6. It could be seen that aposymbiotic insects caused a dramatic decline in the *l*_*x*_ curve and covered a longer age (65 days) when paralleling to that of symbiotic insects. Considering the data in the *s*_*xj*_ curve, the larger slope of the *l*_*x*_ curve in aposymbiotic insects was mainly due to the lower egg hatchability and higher mortality of the 1st and 2nd nymph stages. The age–stage-specific fecundity (*f*_*xj*_), age-specific fecundity (*m*_*x*_), and net maternity (*l*_*x*_*m*_*x*_) of female adults increased first and then decreased with the extension of time (Figure 6). With respect to the *f*_*xj*_ curve, the oviposition in the symbiotic insect started on the 47th day and lasted for 13 days till death, and in the aposymbiotic insect, the oviposition started on the 55th day and lasted for 10 days. However, the top fecundity in the natural group reached 7.3 and the apo-group was 6.1. It is clear aposymbiotic insects caused the oviposition period to shrink and fecundity to fall. It can be referred that the future population of symbiont removal insects would be eliminated. Similarly, the age-specific fecundity (*m*_*x*_) and net maternity (*l*_*x*_*m*_*x*_) covered the same oviposition duration and lower fecundity in aposymbiotic insects. Though the *m*_*x*_ curve showed an exceptionally top fecundity with 4.92 eggs oviposited at day 60, the net maternity (*l*_*x*_*m*_*x*_) was still lower when accompanied by an age-specific survival rate (*l*_*x*_), which was quite low (Figure 6).

**Figure 7:**
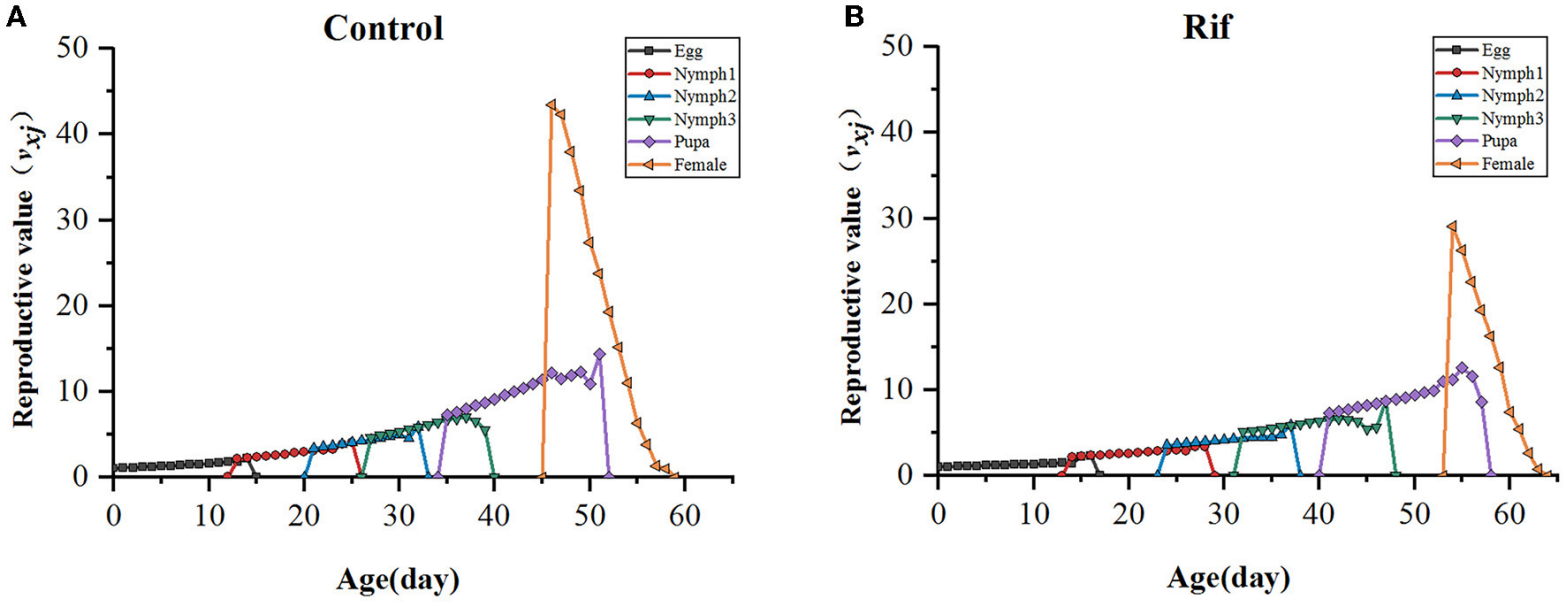
Age–stage-specific reproductive value (*v*_*xj*_) of *A. camelliae*. **(A)** Control, **(B)** Rif-treatment.

In addition, the age–stage-specific reproductive value (*v*_*xj*_) is estimated and illustrated in Figure 7. As data showed, the climbing trend of reproductive value (*v*_*xj*_) with age increasing in the two groups was similar except for the longer stage duration in the aposymbiotic group, and reproductive value reached a maximum around the time of TPOP. For example, the highest reproduction value occurred on the 47th day in the control group, which was close to the day of TPOP (50.29) (Table 4) and *v*_*xj*_ in rif treatment arrived at peak value at day 55 where the TPOP was 57.00 (Table 4). The remarkable difference between both appeared in the adult stage where the top reproductive value of female *A. camelliae* was dropped by 33% in aposymbiotic insects compared with the control group (Figure 7).

#### 3.3.3. Life expectancy of *A. camelliae*

**Figure 8:**
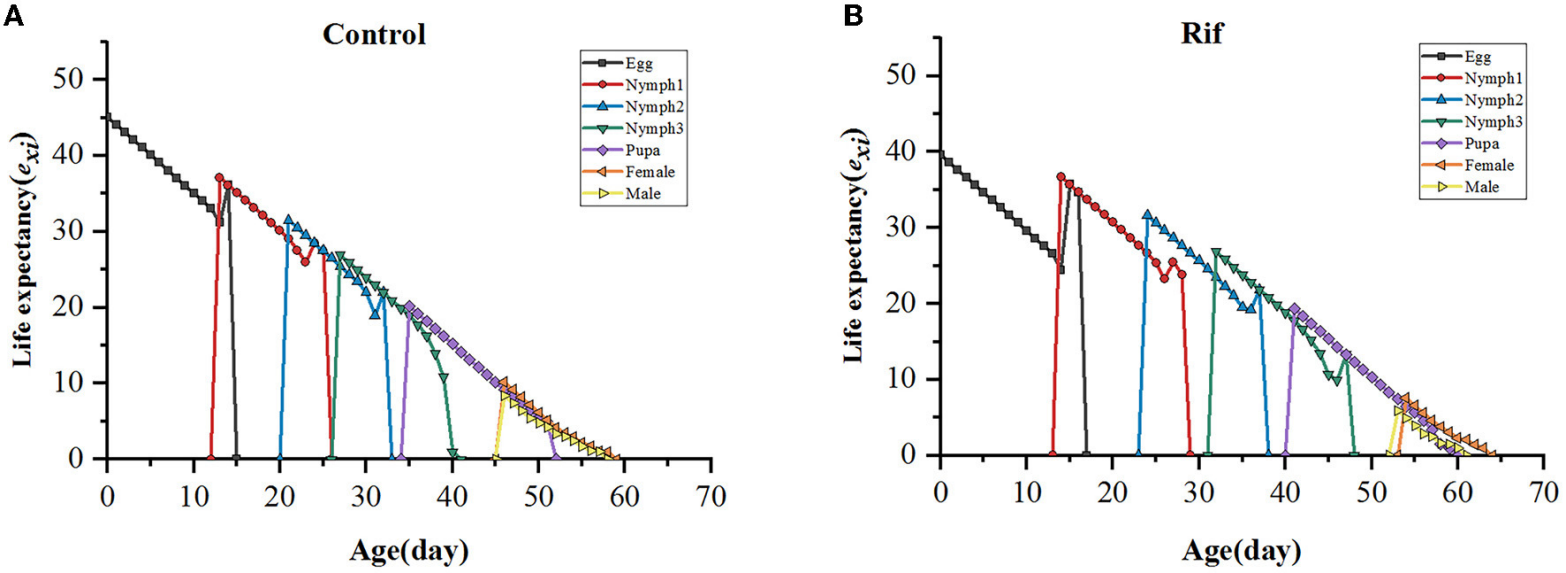
Age–stage life expectancy (*e*_*xj*_) of *A. camelliae* in the control **(A)** and the rif-treatment **(B)**.

The life expectancy (*e*_*xj*_) indicates the length of time that an individual *A. camelliae* of age x and stage j is expected to live. The curve in both groups decreased with age and increased with a degraded level in aposymbiotic insects. The life expectancy of newly hatched eggs of the control and the aposymbiotic insects was estimated to be 45.07 and 39.64 days, respectively (Figure 8).

#### 3.3.4. Life table parameters of *A. camelliae*

The intrinsic rate of increase (*r*) was 0.0279 in the aposymbiotic group that is significantly lower than 0.0446 in the control population. In addition, the net reproductive rate (*R*_0_) and finite rate of increase (λ) of aposymbiotic insects were significantly lower than that of control insects. The mean generation time (*T*) was significantly prolonged in aposymbiotic insects, whereas there was no significant difference in gross reproductive rates (*GRR*) in the two groups of *A. camelliae* (Table 5).

**Table 5:** Population parameters (means ± SE) of *A. camelliae* in the control and the rif-treatment calculated by using the age–stage, two-sex life table.

**Population parameters**	**Control**	**Rif**
Intrinsic rate of increase (*r*) (d^−1^)	0.0446 ± 0.0028a	0.0279 ± 0.0032b
Finite rate of increase (λ) (d^−1^)	1.0456 ± 0.0029a	1.0283 ± 0.0033b
Net reproductive rate (*R_0_*)	10.65 ± 1.55a	5.25 ± 0.98b
Mean generation time (*T*) (d)	53.08 ± 0.33b	59.51 ± 0.30a
Gross reproductive rate (*GRR*)	22.77 ± 2.72a	21.83 ± 2.25a

Standard errors were estimated by using 100,000 resampling. Means followed by different letters are significantly different according to paired bootstrap test based on the confidence intervals of differences between treatments (*P* < 0.05).

## 4. Discussion

Bacterial symbionts play prominent roles in their insect hosts. Many reports related to other whitefly insects, such as *Bemisia tabaci* (Shan et al., 2016; Su et al., 2016; Hashmi et al., 2019; Zhao et al., 2020), proved the effects of symbiotic bacteria on the host growth and development. However, research on symbionts of spiny whitefly and their function was lacking. To make clear this respect, we checked the community composition and diversity of symbiotic bacteria of *A. camelliae* by 16S rRNA high-throughput sequencing and investigated their biological implications by using the age–stage two-sex life table with comparing the aposymbiotic and natural groups.

Our present results proved that the symbiotic bacteria of *A. camelliae* were abundant. Interestingly, it can be observed from the aspect of symbiotic bacteria diversity that larva and adult endosymbiont community diversities did not show obvious changes. We inferred that endosymbionts are in a relatively stable state in *A. camelliae* to mutualism with host insects (Harris et al., 2010). We found that the main five symbiotic bacteria genus were *Portiera, Arsenophonus, Wolbachia, Rickettsia*, and *Pseudomonas*, which together composed approximately 94.83–96.24% in *A. camelliae*. Those type of symbiotic bacteria composition in *A. camelliae* is similar to that in *Bemisia tabaci* (Zchori-Fein et al., 2014; Hashmi et al., 2019) though different in relative content. However, there is an exception that no symbiotic *Hamiltonella* was found in *A. camelliae*, which was reported to exist in *Bemisia tabaci* (Bello et al., 2019). Therefore, we deduced that insects of the same genus have similar symbiotic bacteria.

Proteobacteria are a major group (phylum) of gram-negative bacteria and most important endosymbionts and include a wide variety of bacterial genera in different insects (Yun et al., 2014). It was also found to be dominated in *A. camelliae* in this present result, which was similar to the findings in other Hemipteran insects (Lim and Ab Majid, 2020; Liu et al., 2022). All five main genera, *Portiera, Arsenophonus, Wolbachia, Rickettsia*, and *Pseudomonas*, belonged to the Proteobacteria phylum. Among them, *Portiera* as the primary endosymbiont was the most abundant genera in a stable content level throughout the development cycle of *A. camellia*, indicating that *Portiera* plays a lasting symbiotic role in the growth, development, and survival of *A. camellia*. *Portiera* can evade autophagy of the whitefly bacteriocytes by activating the TOR (target of rapamycin) pathway by providing essential nutrients (Wang et al., 2022). Interestingly, *Portiera* can facilitate the whitefly performance by cooperation with the panBC gene for pantothenate production (Sun et al., 2022). Other secondary endosymbionts' content and relative level would differ among the nymph and adult stages. For example, the relative abundance of *Arsenophonus* (22.89%) was higher than that of *Wolbachia* (4.52%) in the individual larva but lower in the adult stage where *Arsenophonus* accounted for 5.57% and *Wolbachia* increased to 11.58%. This fluctuation suggested a competition between *Arsenophonus* and *Wolbachia* in the growth of *A. camelliae*. This mutual exclusion relationship may be caused by its similar harbor in host cells and competition for limited resources as reported in other insects (Gottlieb et al., 2008; Qu, 2013). Furthermore, the substantial increase in *Wolbachia* level from the larva to adult stages in *A. camelliae* agreed with *Wolbachia* changes in *Diaphorina citri* (Meng et al., 2019) in our early report where *Wolbachia* symbiotic level kept increasing with the growth of tea green leafhopper (Zhang et al., 2022). These results from 16S rRNA sequences were confirmed by the qPCR method. The relative content was consistent in both ways, and all five investigated endosymbionts showed the same relative abundance level and changing tendency from the larva to adult stages in two analysis ways (Figures 2, 4). For example, the *Portiera* content kept at the highest level in both stages (Figure 4A), *Arsenophonus* dropped in adults (Figure 4B), and *Wolbachia* increased (Figure 4C). This indicated that this qPCR result was consistent with Illumina NovaSeq 6000, and the comparison was reliable.

Rifampicin was often applied to achieve aposymbiotic insects to check the change and influence of symbionts (Ahmed et al., 2010; Ren et al., 2021). We also employed rifampicin treatment to perform the symbiotic reduction in *A. camelliae*. We failed to selectively remove secondary symbionts without affecting the primary symbiont in *A. camelliae*. Some articles reported success in selectively removing the secondary symbiont *Hamiltonella* or *Wolbachia* in *B. tabaci*, using the same antibiotic rifampicin without affecting the primary symbiont *Portiera* (Xue et al., 2012; Su et al., 2015). Our results indicated that rifampicin could sufficiently reduce all investigated symbiotic bacteria of *A. camelliae*. Furthermore, *Portiera* was greatly reduced in rif-treatment but inhibited to less extent compared to secondary symbiotic bacteria changes. This phenomenon was also reported in *Bemisia* caused by rifampicin treatment (Shan et al., 2016; Zhao et al., 2020).

The age–stage two-sex life table can help researchers systematically understand the impact of removing symbiotic bacteria on the survival rate, development rate, and reproduction rate of insect populations, as well as the comprehensive impact of various factors on population growth (Chi et al., 2020). We first studied the role of symbiotic bacteria in *A. camelliae* by using the age–stage two-sex life table method. Our result indicated that rif-treatment markedly affected their biological parameters including the net reproductive rate (*R*_0_), intrinsic rate of increase (*r*), and finite rate of increase (λ) (Table 5). Among them, the intrinsic rate of increase (*r*) is a basic ecological feature helpful to estimate the population growth potential (Roy et al., 2003). In our present result, (*r*) was decreased about 2-fold in the rifampicin treatment compared to the control. After symbiotic reduction, this indicated that the preoviposition period (including TPOP and APOP) was prolonged, and the adult longevity was shortened. Moreover, the control group had longer life expectancy and age–stage-specific survival rates in comparison to the rifampicin treatment. These changed biological traits negatively affected the development of *A. camelliae*, ultimately resulting in the reduction of the offspring population. The present results showed that aposymbiotic action had a clear unfavorable influence on *A. camelliae*, which is highly consistent with early reports (Karamipour et al., 2021). For example, bacterial symbiont elimination had negative effects on nymphal development and adult emergence of *Brachynema germari, Acrosternum heegeri*, and *Acrosternum arabicum* (Kashkouli et al., 2019) and adverse effects on life history traits of *Graphosoma lineatum* (Karamipour et al., 2021).

Symbiotic bacteria can help host insects degrade nutrient macromolecules in food and convert them into small molecules that can be directly used to provide missing nutrients for host insects. Endosymbiotic bacteria play an important role in the nutrition utilization of host insects (Frago et al., 2012). Luan et al. (2015) proved that *Portiera*, the primary symbiotic bacteria of *Bemisia tabaci*, was mainly involved in the nutrient metabolism of the host and could provide essential amino acids for the host. Previous studies have shown that *Portiera* may help whitefly improve their performance, and *Portiera* has a close nutritional relationship with their host during host plant acclimation (Hu and Tsai, 2020). The removal of *Arsenophonus* increased the developmental time of the immature stages and reduced the values of different life history parameters in *Ommatissus lybicus* (Karimi et al., 2019). We simply illustrated the function of symbiotic bacteria of *A. camelliae* by metagenomic analysis served by PICRUSt (Supplementary Figure 2D), which allows inference of the functional profile of the symbiotic bacterial consortia. It suggested that metabolism is highly responsible for the relative abundance of function. Whether the effect of symbiotic bacteria of *A. camelliae* is related to the synthesis of amino acids or other respect needs further research. It was needed to mention that rifampicin is broad spectrum and resulted in sufficient changes in symbiont titers of *A. camelliae* and downstream severe fitness defects, including elevated nymphal mortality and reduced population growth parameters. These changes in the population growth may be caused by the collaboration of all consortia of a specific bacterium. However, each specific bacterium and its function could not be specified in this present way. Though it has been reported that rif could specifically reduce *Buchnera* without affecting other bacterial compositions of *Aphis gossypii* (Ayoubi et al., 2020). Classification of the specific bacterium may be realized by the combination of sterilization and infection. On the other hand, this biological change of *A. camelliae* might not only involve bacterial clearance but also other changes in the microenvironment of the host.

## 5. Conclusion

Taken together, we sequenced the *A. camelliae* 16S rRNA gene through the Illumina NovaSeq 6000 platform, which directly revealed the structure of the bacterial community in the larva and adult stages and proved that *Candidatus Portiera, Arsenophonus, Wolbachia, Rickettsia*, and *Pseudomonas* genera of Proteobacteria dominated the whole life cycle of this whitefly. In addition, we confirmed that the rifampicin treatment can suppress the endosymbiont of *A. camelliae* and cause defective host fitness including a longer preadult stage in the offspring generation, a lower survival rate, decreased intrinsic rate of increase (*r*), and net reproductive rate (*R*_0_).

The present study indicated that endosymbionts played an important role in the normal development and growth of *A. camelliae*. Regarding the applied aspect of Camellia spiny whitefly control, the rapid reduction in the primary and secondary symbionts and consequent death of the whitefly hosts indicate that antibiotics or agents targeted on these bacteria may possess the properties of rational insecticides to be used as part of a whitefly management program.

## Data availability statement

The datasets presented in this study can be found in online repositories. The names of the repository/repositories and accession number(s) can be found below: https://www.ncbi.nlm.nih.gov/genbank/, PRJNA910681.

## Author contributions

YT and BG conducted the experiments. YT and JW designed and performed the experiments. YT, QZ, and CL analyzed the data. LJ and XZ conceived and supervised the project. XZ and YT wrote the manuscript. All authors have read and agreed to the published version of the manuscript.
