# Supplementary material for: Diversity of endosymbionts in camellia spiny whitefly, Aleurocanthus camelliae (Hemiptera: Aleyrodidae), estimated by 16S rRNA analysis and their biological implications
Source: Front Microbiol. 2023 Apr 17;14:1124386. doi: 10.3389/fmicb.2023.1124386 (PMC10149810; doi:10.3389/fmicb.2023.1124386)
Supplement: Supplementary file 1 [file Data_Sheet_1.docx]

Supplementary Material

Diversity of endosymbionts in camellia spiny whitefly, *Aleurocanthus camelliae* (Hemiptera: Aleyrodidae), estimated by 16S rRNA analysis and their biological implications

Yanni Tan^1^, Bing Gong^1^, Qiuqiu Zhang^1^, Changkun Li^1^, Junyi Weng^1^, Xia Zhou^1^ and Linhong Jin^1,*^

*** Correspondence:** Linhong Jin: [lhjin@gzu.edu.cn](mailto:lhjin@gzu.edu.cn)

# Supplementary Figures and Tables

## Supplementary Figures

**
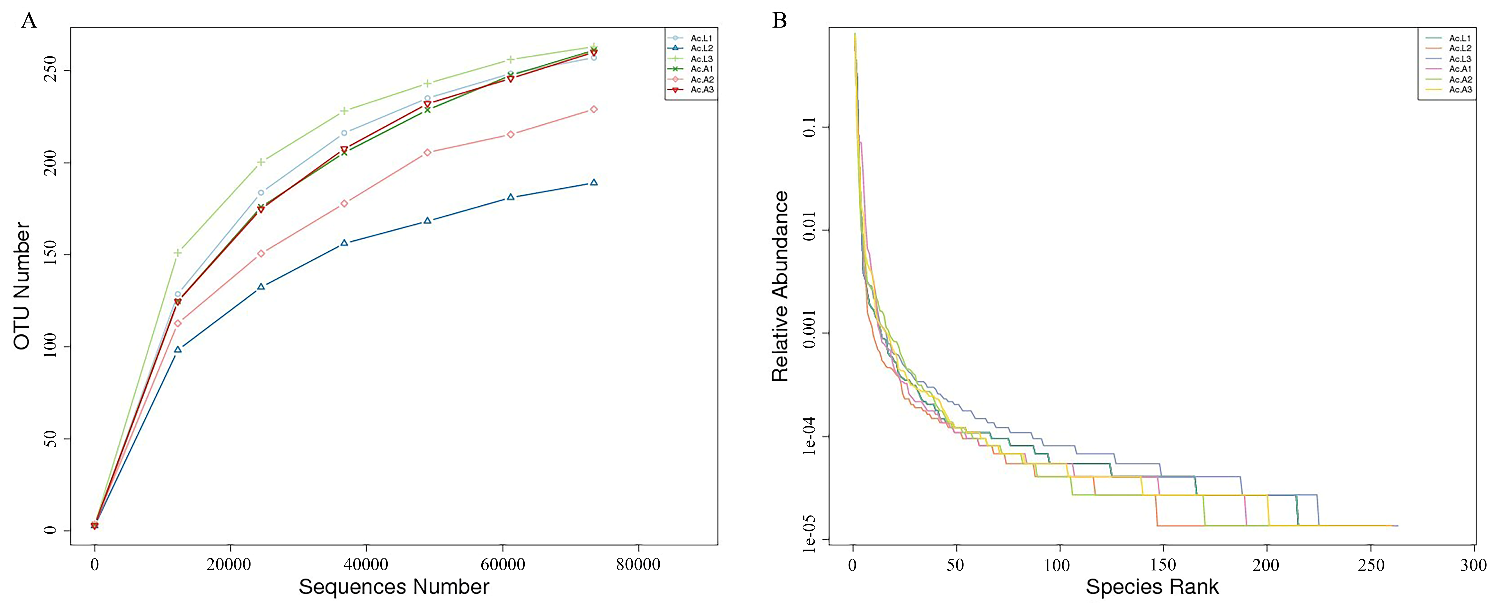
Supplementary Figure 1**. Alpha diversity dilution curve (A) and Rank Abundance curve (B).


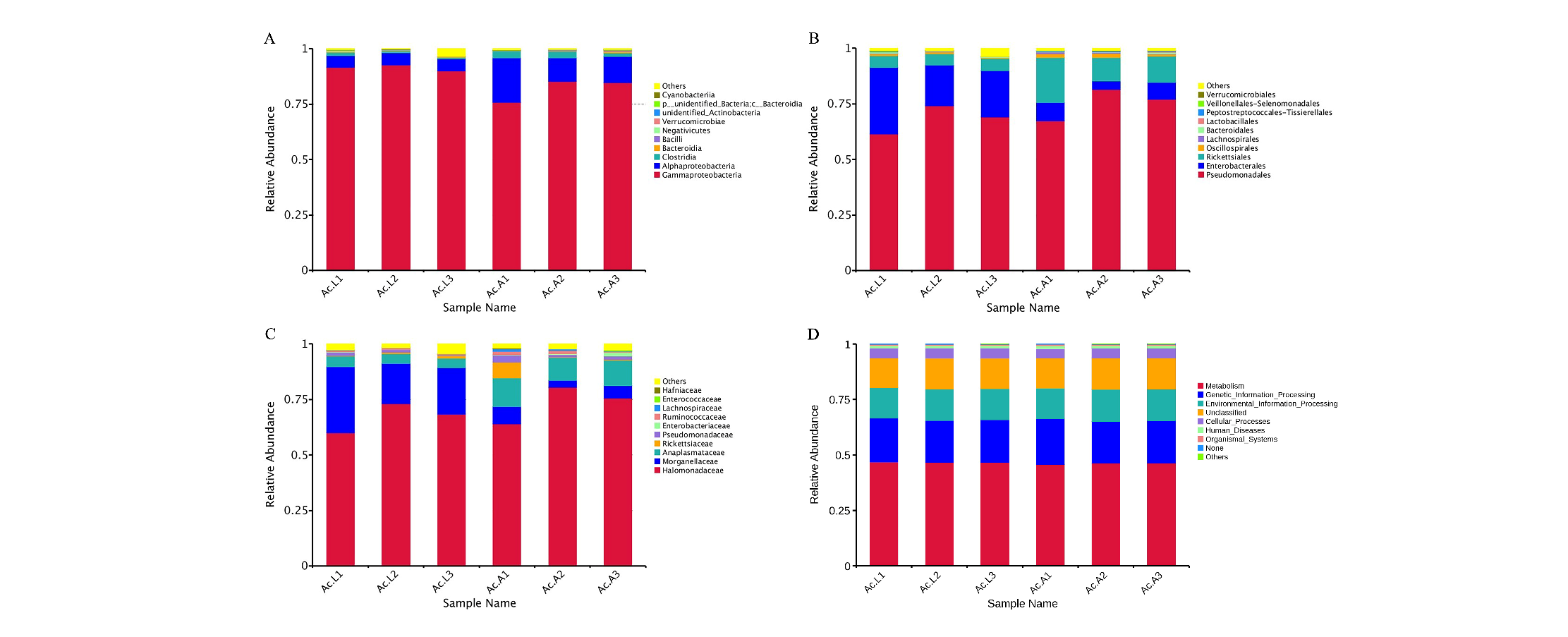


**Supplementary Figure 2**. Bacterial community dynamics among larvae and adults in *A. camelliae* and function prediction. (A) Class; (B) Order; (C) Family; (D) function prediction by PICRUSt.
